# Pentachroma O-H: A Five-Color Histological Staining Method for Enhanced Intestinal Tissue Analysis

**DOI:** 10.3390/ijms262210811

**Published:** 2025-11-07

**Authors:** Emanuel-Ciprian Onica, Cristina-Stefania Dumitru, Flavia Zara, Marius Raica, Cristian Silviu Suciu, Alina Cristina Barb, Oana-Alexia Ene, Cristi Tarta, Dorin Novacescu

**Affiliations:** 1 Department II of Microscopic Morphology, Victor Babes University of Medicine and Pharmacy Timisoara, E. Murgu Square, No. 2, 300041 Timisoara, Romania; onica.ciprian@umft.ro (E.-C.O.); marius.raica@umft.ro (M.R.); cristi.suciu@umft.ro (C.S.S.); toma.alina@umft.ro (A.C.B.); oana.ene@student.umft.ro (O.-A.E.); novacescu.dorin@umft.ro (D.N.); 2 Researching Future Surgery II Research Center, Department X, Discipline of General Surgery II, Faculty of Medicine, Victor Babes University of Medicine and Pharmacy Timisoara, E. Murgu Square, No. 2, 300041 Timisoara, Romania; tarta.cristi@umft.ro

**Keywords:** gastrointestinal tract pathology, morphological diagnosis and prognosis, histological assessment of ileum, original pentachrome stain, gastrointestinal health, gut-associated lymphoid tissue immune cells, histochemistry biomarkers, mucosal epithelium, improved methodology for pathological evaluation in gastroenterology

## Abstract

Current histological staining methods for intestinal tissue analysis face limitations in simultaneously visualizing multiple tissue components, often requiring multiple sequential stains that increase processing time and tissue consumption. This proof-of-concept study aimed to define and develop a pentachromatic staining method for enhanced visualization of gastrointestinal tissue architecture. We developed the Pentachroma O-H method, an original protocol using readily available histological reagents (Alcian Blue pH 2.5, Weigert’s resorcin–fuchsin, Mayer’s hematoxylin, and Van Gieson’s solution) applied in an optimized sequence. The protocol was tested on healthy human ileum tissue obtained from surgical specimens as proof of concept. Thirty serial sections were stained with Pentachroma O-H and compared to adjacent sections stained with conventional hematoxylin–eosin (H&E) to document the emerging morphological characteristics of this original stain. Pentachroma O-H achieved distinct five-color differentiation in approximately 45 min: acidic mucins appeared turquoise–blue, collagen fibers red, elastic fibers black–purple, smooth muscle and erythrocyte cytoplasm yellow, and nuclei blue–black. The method clearly delineated intestinal architecture, including mucosal goblet cells, muscularis mucosae, connective tissue vasculature (parietal smooth muscle and elastic laminae), fibers, and cellular components, as well as lymphoid tissue aggregates and infiltrates, with improved contrast compared to H&E. All tissue components were simultaneously visualized in single sections with excellent morphological preservation. This first description of Pentachroma O-H demonstrates its capability to provide comprehensive ileum tissue visualization equivalent to multiple traditional special stains in a single, efficient protocol, offering significant potential advantages for gastrointestinal pathology assessment and warranting future validation studies across diverse tissue types and pathological conditions.

## 1. Introduction

Histological examination of intestinal tissue remains a cornerstone of gastrointestinal pathology, essential for diagnosing inflammatory bowel disease, neoplastic conditions, and various malabsorption disorders [1]. However, current staining methodologies face significant limitations in providing comprehensive intestinal tissue visualization necessary for accurate diagnosis and research applications [2]. Thus far, diagnostic protocols still require multiple tissue sections with different targeted stains, leading to increased processing time, tissue consumption, and potential inter-section variability [2], thereby emphasizing the need for more efficient combined protocols.

The intestinal mucosa presents particular challenges for histological analysis due to its complex architecture comprising an epithelial mucosa with abundant goblet cells, a lamina propria, submucosa, and adventitia, each rich in various connective tissue components, and multiple smooth muscle layers [3]. Inflammatory bowel diseases, which affect millions worldwide, require precise assessment of mucosal architecture, inflammatory infiltrates, fibrosis, and goblet cell population, for accurate diagnosis and treatment monitoring [4].

Histological staining techniques are fundamental for revealing tissue morphology and structure in diagnostic pathology. The traditional hematoxylin and eosin (H&E) stain, while universally employed, provides only basic nuclear–cytoplasmic contrast and morphological information, visualizing nuclei in blue–purple and cytoplasm/fibers in shades of pink [5]. However, important components like mucus and elastic fibers can be difficult to discern with H&E alone—mucin is easily washed out during tissue processing, resulting in very poor staining with routine H&E, whereas collagen versus smooth muscle both take up eosin, albeit at different intensities, staining in chromatically similar acidophilic tones (pink-reddish), which at times may provide poor contrast. This limited palette proves insufficient for the complex architectural assessment required in intestinal pathology, where simultaneous visualization of mucins, elastic fibers, collagen, smooth muscle, vasculature, and lymphoid tissue is crucial for comprehensive evaluation [6]. Thus, beyond the routine H&E stain, a variety of special histochemical and immunohistochemistry (IHC) stains have been developed to highlight specific tissue components. 

Current multi-component staining methods, such as Masson’s trichrome and Movat’s pentachrome, offer improved tissue differentiation but remain suboptimal for intestinal tissue analysis. Masson’s trichrome, for example, uses three dyes to differentially stain muscle, collagen, fibrin, and erythrocytes—classically producing black nuclei, red muscle/cytoplasm, and blue (or Gomori green) collagen fibers [7,8]. This trichrome technique is routinely used to assess fibrosis (excess collagen) or to distinguish smooth muscle in tumors [7]. However, it does not specifically identify mucins or elastic fibers. 

Periodic acid–Schiff (PAS) is another widespread method, which stains glycogen and other polysaccharides magenta by reacting periodic acid-generated aldehydes with Schiff’s reagent [8]. PAS highlights neutral mucins, basement membranes, and fungal cell walls, among other carbohydrate-rich structures [8]. It is often combined with diastase digestion to confirm glycogen, or with Alcian Blue as in the Alcian Blue–PAS (AB/PAS) dual stain to differentiate acidic versus neutral mucins on one slide [9]. In the AB/PAS method, Alcian Blue (at pH 2.5) first stains acidic mucins blue; then, PAS yields magenta coloration of neutral mucins, allowing both types of mucus to be visualized distinctly [9]. While PAS is very versatile for carbohydrates, it does not impart contrast to collagen or muscle fibers.

Alcian Blue itself is a cationic copper phthalocyanine dye that binds strongly to anionic polysaccharides like glycosaminoglycans and acidic mucins [10]. It selectively stains acidic epithelial and connective tissue mucins blue to turquoise, a property widely exploited in histology [10]. Alcian Blue at pH 2.5 will mark both sulfated and carboxylated mucins (e.g., intestinal goblet cell mucins), turning those tissue components a distinctive blue–green hue, often termed alcianophilic [11]. This stain is especially useful for identifying mucin-secreting cells and is commonly applied in gastrointestinal and mucin-producing tumor pathology. However, on its own it provides no information on collagen, muscle, or elastic fibers.

Toluidine Blue is a basic thiazine dye known for its metachromasia: it stains nuclei and background structures blue, but certain substances with high acidic polyanion content (like mast cell granules or cartilage matrix proteoglycans) shift the dye’s color to purple–red [12]. Toluidine Blue is frequently used to rapidly screen mast cells (whose heparin-rich granules show purple metachromasia) and to highlight cartilage or mucins in tissues [12]. While it increases histologic detail and can stain virtually all structures in thin resin sections, its color range is limited (essentially blue vs. purplish shades), and it cannot distinctly resolve multiple tissue components by unique colors—many elements other than mast cell granules will simply appear in shades of blue. Thus, toluidine blue’s diagnostic utility is more restricted (e.g., quick intraoperative frozen staining or specific identification of mast cells [12]) compared to multi-color histochemical methods.

To visualize complex tissue architecture with greater clarity, pathologists often employ combinatorial stains or sequential use of multiple special stains. Notably, the combination of Verhoeff’s iron hematoxylin and Van Gieson’s picric acid/fuchsin (the Verhoeff-Van Gieson stain) colors elastic fibers black and collagen fibers red against a yellow background (cytoplasm and muscle) [13]. Building of the premises, complex pentachromic combinations of staining reagents were investigated, yet only scarce pathological tools managed to be translated into clinical practice. Movat’s pentachrome is the main classic example that achieves a five-color differentiation on a single slide [14]. 

Originally developed by Movat in 1955 and later modified by Russell in 1972, Movat’s stain uses a sequence of reagents to highlight connective tissue constituents in five distinct hues [14,15]. In its modified form, it employs Alcian Blue for acidic mucins, Verhoeff’s iron hematoxylin for nuclei and elastic fibers, and a mixture of crocein scarlet and acid fuchsin for muscle (and fibrin), followed by phosphotungstic acid and saffron to stain collagen [15]. The result is a strikingly detailed palette: elastic fibers and nuclei are stained black, mucins and ground substance blue, muscle red, fibrin intense red, and collagen yellow [16]. This comprehensive staining reveals histologic relationships—used specifically for cardiovascular tissues, one can simultaneously see elastin fragmentation, intimal mucin deposition, muscle layers, and collagenous fibrosis in a single section [17]. 

Movat’s pentachrome has proven valuable for diagnosing vascular diseases and cardiac pathology [17,18]. However, the method is technically complex and time-consuming; the original protocol required extended incubation and multiple differentiation steps, prompting Russell’s modifications to improve consistency and reduce staining time [15]. Even so, Movat’s stain involves handling of multiple reagents (including caustic or hazardous chemicals like ferric chloride and the natural dye saffron) and careful timing to avoid unwanted background staining. These practical challenges, along with the expense or limited availability of certain reagents (e.g., saffron), have limited its routine use in many pathology labs.

Given the limitations of current histological stains in visualizing complex tissue architectures, there is a clear need for a new multipurpose staining method that can provide broad contrast without undue complexity. To sum up, existing stains like Masson’s or Van Gieson’s trichrome differentiate collagen from muscle, or AB-PAS distinguishes mucin types, but no single traditional stain (aside from Movat’s) simultaneously delineates mucins, collagen, elastic fibers, and muscle in one preparation. In practice, pathologists may have to order two or three different special stains on serial sections to piece together a diagnostic picture—for example, an AB/PAS for mucus, a trichrome for fibrosis, and an orcein stain for vascular elastin. Each special stain indeed provides targeted information, but performing multiple sequential stains on separate sections makes correlating findings more challenging (since the features are on separate slides), is time-consuming, and wastes precious tissue, especially in small biopsies. A unified pentachromatic approach could address these issues by revealing multiple key tissue constituents in situ together.

This study presents the development and validation of “Pentachroma O-H,” an original five-color staining method designed to improve tissue contrast and element-specific visualization in routine histology, focusing on ileum tissue analysis. The name reflects its pentachromatic nature and the initials of its creator and origin (Onica Emanuel-Ciprian, our staining technician at the Histology Discipline, UMFVB Timisoara). The goal of Pentachroma O-H is to provide a single-slide solution that highlights acidic mucins, collagenous connective tissue, elastic fibers, smooth muscle, and nuclei with distinct colors. In doing so, it aims to enhance diagnostic precision—for instance, by making subtle mucin deposits or early fibrous changes readily apparent in the context of overall tissue architecture, which might be missed or deemphasized on H&E. Improved contrast can be clinically significant in gastrointestinal pathology: in diseases such as ulcerative colitis or certain adenocarcinomas, the presence and distribution of mucin alongside fibrosis or elastin changes can influence diagnosis and grading. Similarly, in cardiovascular pathology, detecting elastic fiber breaks or intimal mucin accumulation early could aid in diagnosing arteriosclerosis or connective tissue disorders.

By developing a robust yet straightforward pentachrome stain, using commonly available reagents (Mayer’s hematoxylin, Weigert’s resorcin–fuchsin, and Van Gieson’s picric acid–fuchsin), we also emphasize practical workflow integration, while providing comprehensive tissue visualization in a single, efficient protocol. The Pentachroma O-H method is optimized for standard formalin-fixed, paraffin-embedded (FFPE) tissues and can be completed in roughly an hour, making it feasible for routine use, analogous to other special stains. In the following, we detail the staining protocol and reagents, compare the histological results on normal human ileum tissue versus conventional H&E, and discuss the implications of this method relative to existing techniques.

## 2. Results

### 2.1. Optimization of Staining Sequence

Initial experiments tested various sequences of the four staining components to determine the optimal protocol. The sequence proved critical for preventing color interference and achieving maximum contrast between tissue elements. The optimal sequence was established as Alcian Blue → Weigert’s elastin → Mayer’s Hematoxylin → van Gieson. This order prevented masking of specific components and color bleeding between adjacent structures that occurred with alternative sequences. When the elastin stain was applied before Alcian Blue, for instance, the mucin staining was significantly diminished. Similarly, applying van Gieson before the nuclear stain resulted in poor nuclear definition due to acid interference with hematoxylin binding.

### 2.2. Color Differentiation and Tissue Component Visualization

**Figure 1:**
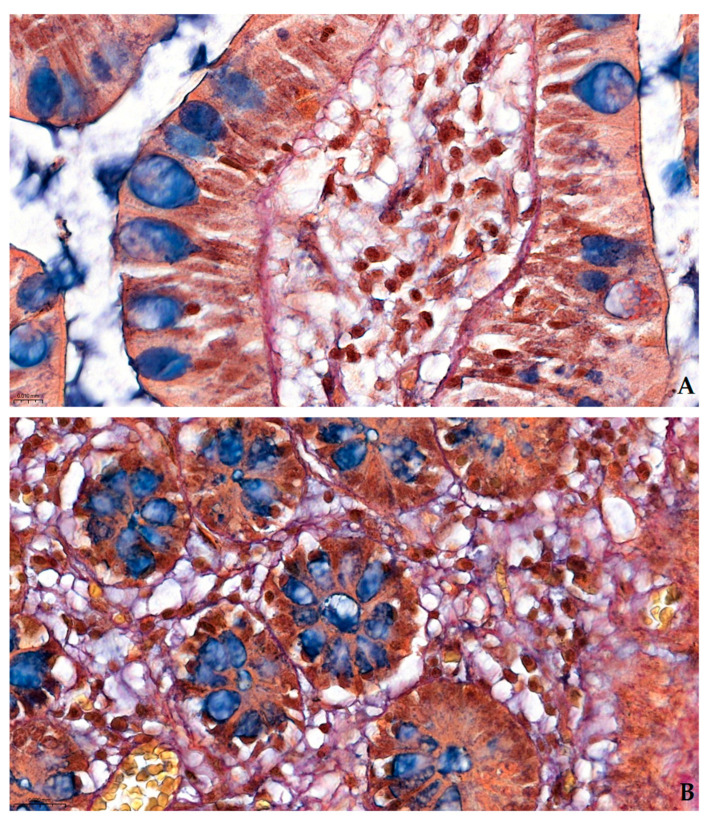
Pentachroma O-H staining of human ileum mucosa at high magnification (400×)—Comparative sections: (**A**) longitudinal section through an intestinal villus showing surface epithelium composed of absorptive enterocytes with basal blue–black nuclei (Mayer’s hematoxylin) and scattered goblet cells, whose acidic mucins are highlighted in turquoise–blue (Alcian Blue pH 2.5). The lamina propria core contains capillaries, connective tissue, and scattered elastic fibers; (**B**) transverse section through adjacent villi at the same magnification, revealing the circular arrangement of epithelial cells, with centrally located goblet cells, rich in acidic mucins (turquoise–blue), and a surrounding stroma, composed of red-stained collagen (Van Gieson’s acid fuchsin), yellow-to-orange cytoplasmic (i.e., erythrocytes) and muscular elements (picric acid), and black–purple elastic fibers (Weigert’s resorcin–fuchsin) distributed within the lamina propria. Scale bar: (**A**)—0.010 mm; (**B**)—0.020 mm.

**Figure 2:**
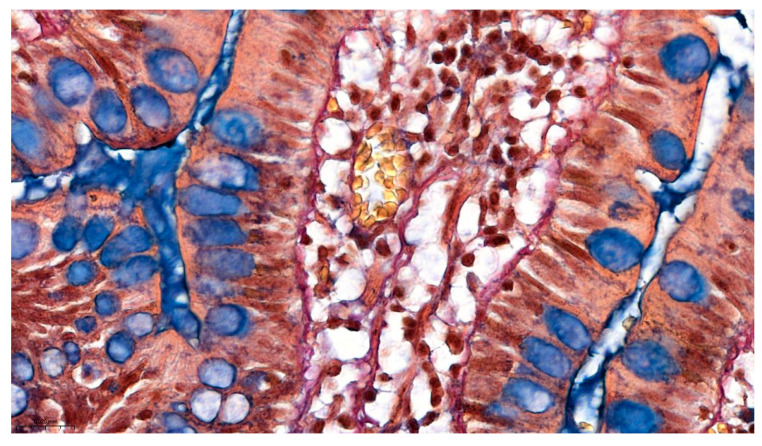
Details of human ileum villus on longitudinal section, at high magnification (400×), in Pentachroma O-H staining: surface epithelium—absorptive enterocytes (Mayer’s hematoxylin stains basal nuclei blue–black, yet, at times, depending on sectioning, overlapping cytoplasmic reactivity may hinder visualization—more visible in the mucosa on the left side of the villus) and scattered goblet cells (Alcian Blue pH 2.5 stains acidic mucins turquoise–blue), sitting on a dark red-to-purple basement membrane (Van Gieson’s acid fuchsin stains collagen red, while Weigert’s resorcin–fuchsin stains elastic fibers dark purple) and a central stromal axis, with capillaries (picric acid stains the cytoplasm yellow–orange in erythrocytes), connective tissue fibers (scarce red collagen and more abundant dark purple elastin), and scattered mesenchymal/immune cellularity (blue–black nuclei). Scale bar—0.020 mm.

Pentachroma O-H produced vivid multicolor differentiation of all the targeted ileum tissue structures, clearly visualizing epithelial, stromal, lymphoid, and vascular components concomitantly, within the same section (see Figure 1 and Figure 2).

#### 2.2.1. Comparative Sections Through Ileum Villi

At a high magnification (400×), in a longitudinal section through the intestinal villus (see Figure 1A and Figure 2), at the level of the ileum mucosa, the absorptive enterocytes lining the villus display, at times, crisp blue–black basal nuclei due to Mayer’s hematoxylin, whereas the abundant goblet cells are readily identified by their turquoise–blue apical mucin granules (Alcian Blue solution, pH 2.5). These stained goblet cell mucins stand out against the neighboring enterocytes, indicating acidic mucosubstances that would be nearly invisible in H&E, due to wash-out during processing. Beneath the mucosa, a well-highlighted basement membrane is clearly discernable, creating a diffusion interface between the overlaying epithelium and the subjacent loose connective tissue (see Figure 1A).

Conversely, Figure 1B, a transverse section through adjacent villi, highlights the circumferential arrangement of the epithelial layer encircling each villus. Central goblet cells filled with blue alcianophilic mucin punctuate the epithelial rosettes, while the surrounding lamina propria also shows, as described above for the longitudinal section, the full array of Pentachroma O-H-specific connective tissue interactions: scarce red collagen fibers in the villus core (acid fuchsin); distinct golden–yellow vascular smooth muscle and yellow-to-orange erythrocyte cytoplasmic elements (picric acid); and thin stromal black–purple strands of elastin (resorcin–fuchsin).

**Figure 3:**
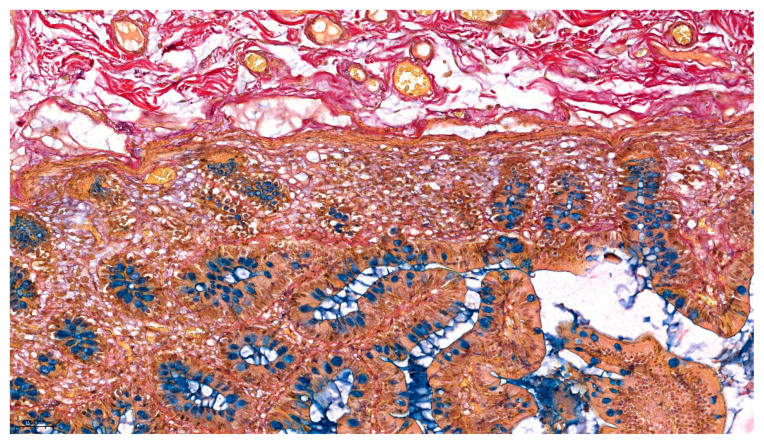
Overview of the mucosal architecture of the human ileum in Pentachroma O-H staining: mucosa and submucosa at 100× magnification. The intestinal villi are composed of columnar absorptive epithelium and goblet cells with blue-stained acidic mucins (Alcian Blue), distributed across the mucosal surface. The underlying lamina propria and sub-mucosa display abundant collagen fibers in red (acid fuchsin, Van Gieson), interspersed with elastic fibers stained dark purple (Weigert’s resorcin–fuchsin). Smooth muscle elements of the muscularis mucosae and vasculature are visualized in yellow (picric acid), while cell nuclei appear blue–black (Mayer’s hematoxylin). Scale bar—0.060 mm.

Figure 2 highlights the delicate lamina propria core of the villus, exemplifying the complete pallet of constitutive elements for loose connective tissue: capillary erythrocytes appear yellowish, the scarce collagenous matrix is stained bright red, and the more abundant fine elastic fibers are stained in a black–purple hue. The red coloration corresponds to acid fuchsin binding to collagen fibrils, while the black–purple elastic fibers reflect the uptake of Weigert’s resorcin–fuchsin. Scattered lymphocytes and other stromal cell nuclei were also tinted blue–black (with Mayer’s hematoxylin). This multicolor contrast allows individual tissue constituents to be distinguished at a glance—for example, an elastin fiber can be traced coursing between collagen bundles, and a thin muscle fascicle at the villus base is discernable by its yellow tone amid the red collagenous background (see Figure 3).

#### 2.2.2. Organ Architecture, Vasculature and Lymphoid Structures

Our combined histochemical staining protocol maintained excellent tissue morphology with minimal artifacts throughout all sections. The intestinal villi remained intact with clear visualization of the epithelial “brush border” (see Figure 1 and Figure 2). Crypts of Lieberkühn showed well-preserved architecture with distinct stem cell zones and differentiated cell populations (see Figure 3). The lamina propria of the mucosa, containing both collagen and cells, appeared as a reddish stippled area with embedded blue nuclei and capillaries. This preservation of fine morphological detail was consistent across all tissue components, enabling reliable interpretation of both normal architecture and pathological changes.

At a low magnification, the Pentachroma O-H method provided an informative overview of ileum tissue architecture that could not have been achieved within a single conventional H&E slide. Figure 3 illustrates an ileum section stained with Pentachroma O-H at 100×, encompassing the mucosa and submucosa. The tall villi are tipped with a discontinuous band of blue–turquoise, corresponding to the acidic mucins in goblet cells distributed along the epithelium, while the underlying lamina propria (albeit less fibrillary—loose connective tissue) and submucosa (dense irregular connective tissue) demonstrate a complex network of connective fibers, with dense red collagen bundles (the bulk of the connective tissue scaffold, well expressed in the submucosa), interwoven with thin threads of fine dark purple elastin fibers. This multicolor contrast delineates the boundary between mucosa and submucosa and can highlight areas of fibrosis or elastosis that would appear homogeneous on H&E. Notably, smooth muscle elements were also simultaneously highlighted in Pentachroma O-H sections, i.e., the muscularis mucosae and the walls of small blood vessels in the submucosa were stained distinctly yellow. This yellow tint, imparted by picric acid, cleanly differentiated muscle fibers from adjacent red collagen fibers. Even at the overview level, the nuclei of epithelial, stromal, and smooth muscle cells retained a distinctive blue–black counterstain throughout the field, providing sharp definition of cell density and localization, while ensuring that tissue architecture and cellular detail remained interpretable. Such comprehensive staining allows the observer to easily and accurately distinguish the mucosal layer from the submucosa (which is rich in larger blood vessels and connective tissue, beneath the yellow muscularis mucosae) and to appreciate the integrity of the muscularis mucosae and blood vessel walls within the submucosa.

**Figure 4:**
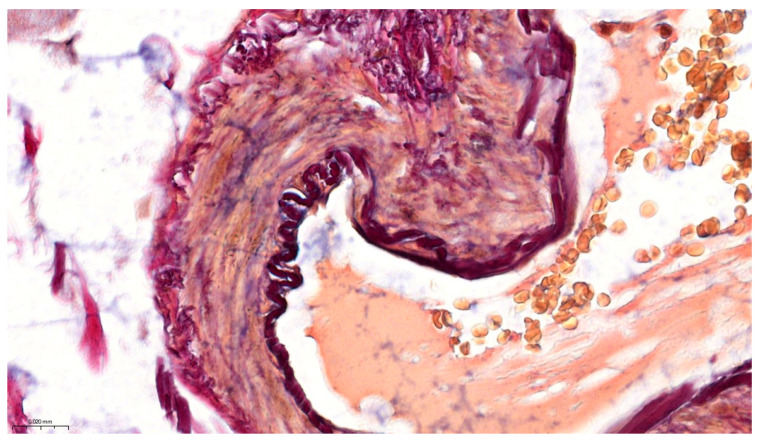
Vascular architecture details of the human ileum in Pentachroma O-H staining: high-power image (400×) of a small arteriole within the ileum submucosa. The wall of the vessel demonstrates concentric layers of smooth muscle in the tunica media stained yellow (picric acid), surrounded externally by red-stained collagen (acid fuchsin). The internal elastic lamina is distinctly visible as a sharply delineated black–purple band (resorcin-fuchsin), clearly separating the intimal layer from the muscular wall. The vessel lumen contains erythrocytes with a yellow–orange cytoplasm, while endothelial and stromal nuclei are blue–black. Scale bar—0.020 mm.

Pentachroma O-H proved particularly advantageous for visualizing vascular structures. Figure 4 focused on a detail of a small submucosal arteriole at 400×. The arterial wall’s architecture was vividly delineated: the concentric smooth muscle layers of the tunica media, encircling the lumen, were stained bright yellow, being neatly demarcated from the surrounding peripheral collagen in the outer tunica adventitia, which was stained red. At the interface between these layers, the internal elastic lamina became sharply visible as a thin black–purple band encircling the intima, thanks to the resorcin–fuchsin’s affinity for elastin. This level of detail—particularly visualizing the elastic lamina as a clear distinctly stained arterial wall structure—is rarely, if ever, achievable on a routine H&E sections, yet our Pentachroma O-H rendered it consistently, with remarkably high contrast. Erythrocytes within the arteriole lumen appeared in yellow–orange tones (picric acid staining of hemoglobin-rich cells), and the endothelial cell nuclei lining the vessel (tunica intima), as well as fibroblast nuclei in the vessel’s outer connective tissue, were clearly stained blue–black. These amounting details confirm that our original multicolor stain can concurrently highlight mucosal, stromal, and vascular components across different magnifications (Figure 3 and Figure 4), thus allowing for simultaneous assessment of vascular smooth muscle integrity (yellow media), elastic fiber distribution (purple–black lamina), and perivascular collagen (red adventitia), all in one view. Such detailed visualization can aid in evaluating pathological ileum changes like fibrosis or elastosis in vessels and stroma without relying on multiple separate stains.

**Figure 5:**
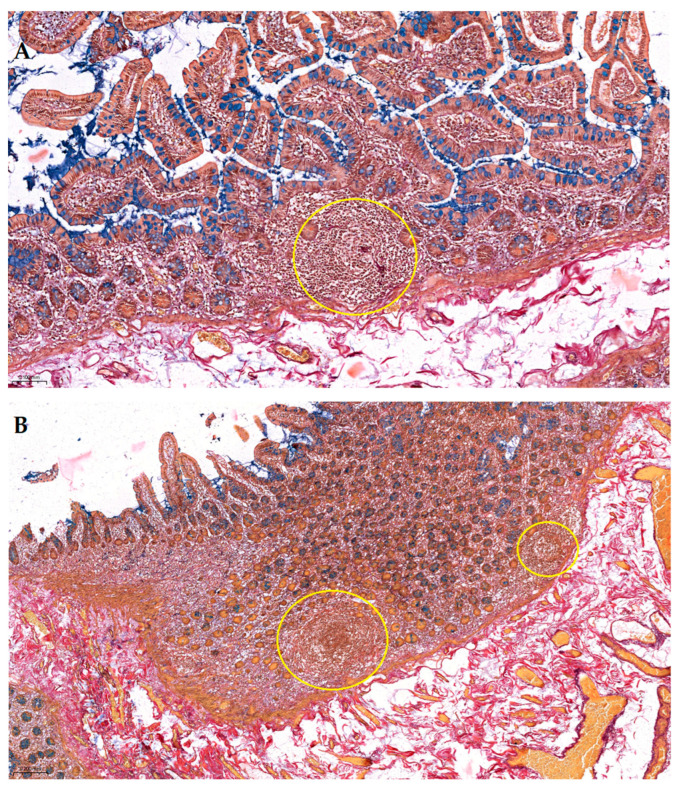
Sections of human ileum in Pentachroma O-H, illustrating the morphology of Peyer’s patches (yellow circles), at different magnifications: (**A**) Transition between mucosa and submucosa (100×)—Intestinal villi in longitudinal and transverse sections, showing typical architecture of the intestinal mucosa: columnar absorptive epithelium and goblet cells with acidic mucins, distributed across a basement membrane, with a central loose connective tissue axis (lamina propria) and a clearly highlighted muscularis mucosa (at the interface between the mucosa, i.e., the loose connective tissue of the lamina propria, and the submucosa, i.e., dense irregular connective tissue). Centrally, just above the muscularis mucosa, a Payer’s patch is visualized at the base of the mucosa, extending upward into the lamina propria, i.e., well-organized gut-associated lymphoid tissue (GALT): B-cell-rich germinal centers within the aggregated follicles and T-cell-rich parafollicular zones between them. Comprised mainly of lymphocytes, with scant cytoplasm, and very scarce follicular stroma, this patch appears as a pale area with dense cellularity. Lymphoid cell nuclei appear blue–black (Mayer’s hematoxylin), condensed towards the center of the patch, while the peripheral border is faded, without a clear distinction of the follicle-associated epithelium (FAE). (**B**) Wide overview (50×) of the transition between mucosa and submucosa—Architectural elements remain pervasive and clearly visible: the same consistent staining patterns for the mucosa (epithelium lining, lamina propria, and muscularis mucosa) and subjacent submucosa (extensive red collagen matrix, intertwined with dark purple elastin fibers, holding an extensive and distinctively yellow-stained vascular network). At the level of this mucosa-to-submucosa interface, sitting just above the muscularis mucosa, two neighboring Payer’s patches can be clearly seen, extending upward into the lamina propria (poorly bordered pale areas with numerous blue–black nuclei). Scale bar: (**A**)—0.100 mm; (**B**)—0.200 mm.

Lastly, Pentachroma O-H staining also effectively demonstrated gut-associated lymphoid tissue (GALT) morphologies. Figure 5A shows a Peyer’s patch at 100× magnification, positioned just above the muscularis mucosae and extending upward into the lamina propria. The lymphoid aggregate appears as a pale area with densely packed blue–black nuclei (Mayer’s hematoxylin), representing the B-cell-rich germinal centers and T-cell-rich parafollicular zones. The sparse cytoplasm and minimal follicular stroma result in limited uptake of the other stains, creating a characteristic pale appearance against the surrounding colorful connective tissue. The peripheral border of the Peyer’s patch fades gradually without a clearly defined follicle-associated epithelium (FAE). Figure 5B provides a wider overview at 50× magnification, demonstrating two neighboring Peyer’s patches at the mucosa–submucosa interface, both showing the same characteristic pale appearance with numerous condensed blue–black nuclei, clearly demarcated from the extensively red collagen matrix and yellow muscularis mucosa/vascular network of the submucosa.

**Figure 6:**
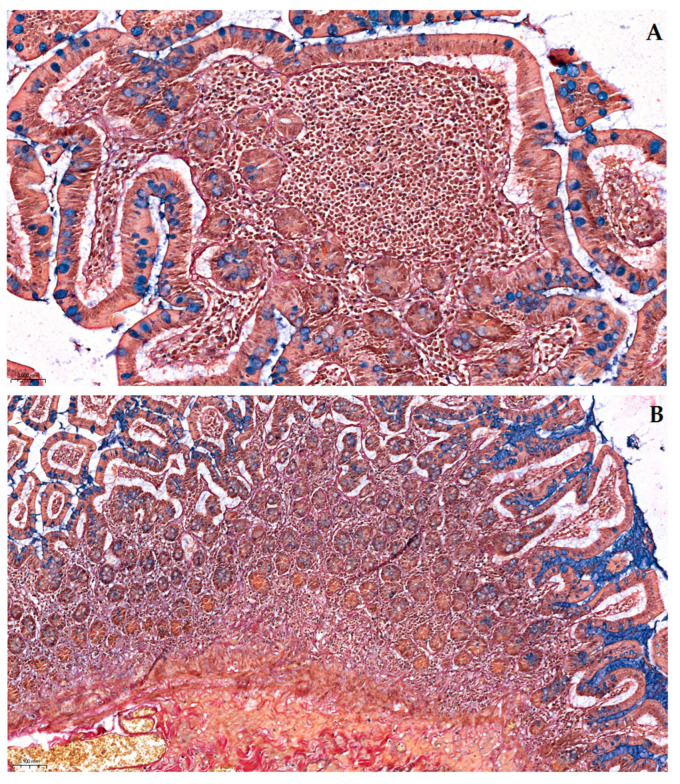
Sections of human ileum in Pentachroma O-H, illustrating the morphology of mucosal lymphoid infiltration of the mucosa, at different magnifications: (**A**) Intestinal villi in mixed longitudinal/transverse sections (200×), showing typical intestinal epithelium, circumscribing heavily augmented lamina propria cores: scarce loose connective tissue stroma with extensive lymphoid infiltration and immune cell accumulation/aggregation. The same blue–black nuclear tinctoriality is maintained throughout the invasive immune cellularity. (**B**) Overview (100×) of the transition between mucosa and submucosa, with the same architecture and tinctoriality as previously described, yet showing a denser cellularity in the lamina propria, due to abundant lymphoid infiltration: numerous blue–black nuclei, clustered within a diminishing loose connective tissue stroma, overwhelmed by immune cell accumulation. Scale bar: (**A**)—0.060 mm; (**B**)—0.100 mm.

Beyond organized lymphoid tissue, the Pentachroma O-H method also revealed diffuse lymphoid infiltration within the mucosa. Figure 6A (200×) demonstrates intestinal villi with heavily augmented lamina propria cores, showing extensive lymphoid infiltration and immune cell accumulation. The infiltrating lymphocytes maintain the same blue–black nuclear tinctoriality throughout, creating dense cellular aggregates within the diminishing loose connective tissue stroma. Figure 6B (100×) provides an overview of this lymphoid infiltration pattern across multiple villi, with numerous blue–black nuclei clustered within the lamina propria, overwhelming the normal stromal architecture.

**Figure 7:**
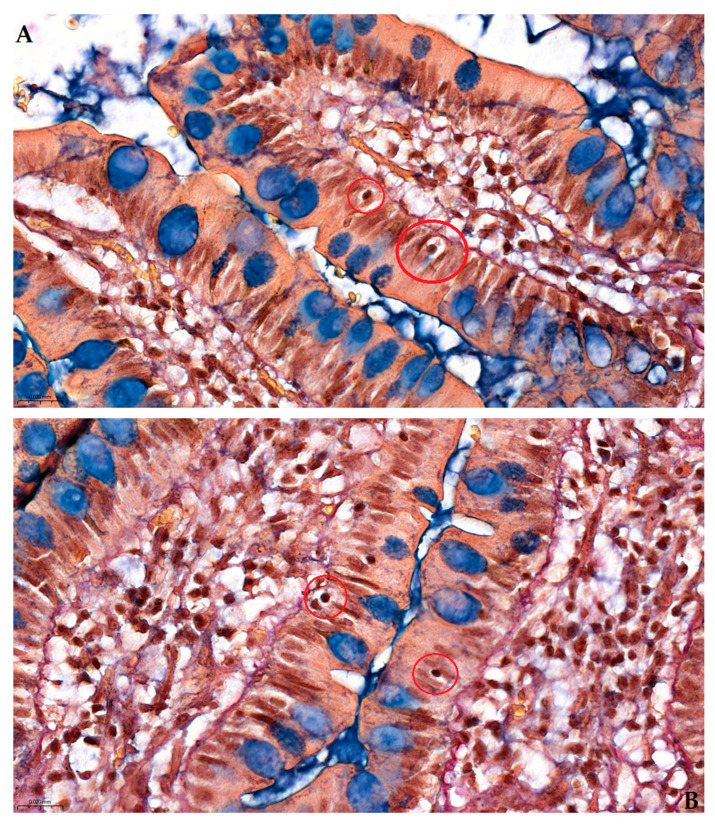
High-magnification (400×) images of longitudinal villous sections through human ileum in Pentachroma O-H, focusing on intraepithelial lymphocytes (red circles), alongside cellularity details of the epithelial “brush-border”: (**A**) two clearly visible neighboring intraepithelial lymphocytes within the same villus, with their characteristic perinuclear clearing (or halo), located at the base of the epithelial ileum lining, just above the basement membrane; and (**B**) a comparative example of intraepithelial lymphocytes, with the same aforementioned pathognomonic characteristics, yet within two distinct neighboring parallel villi. Scale bar: (**A**)—0.020 mm; (**B**)—0.020 mm.

The method’s resolution extends to individual cellular details, including intraepithelial lymphocytes. Figure 7A (400×) clearly demonstrates two neighboring intraepithelial lymphocytes within a single villus, displaying their characteristic perinuclear clearing (halo) and location at the base of the epithelial lining, just above the basement membrane. Figure 7B shows a comparative example from adjacent parallel villi, confirming the consistent visualization of these diagnostically important cells with their pathognomonic morphological features.

### 2.3. Direct Side-by-Side Comparison with Conventional Staining Morphology

Direct side-by-side comparison with sequential H&E staining on serial sections confirmed several striking advantages in favor of our original pentachromatic stain (i.e., superior contrast, enhanced histological visualization, better appreciation of tissue component interactions, improved specificity, and superior characterization of spatial relationships).

**Figure 8:**
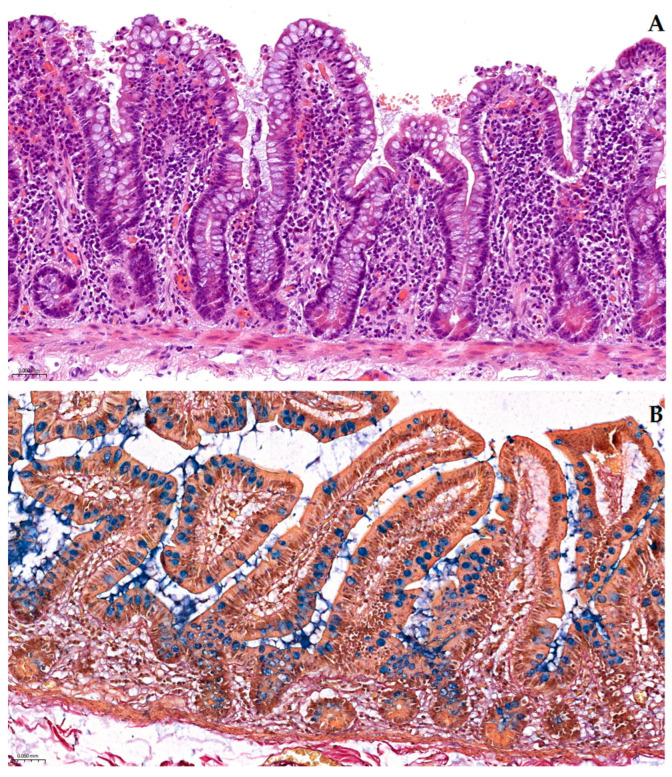
Comparative staining of human ileum mucosa at 200× magnification: (**A**) Classical hematoxylin–eosin (H&E) stain showing intestinal villi in longitudinal section, with a predominance of absorptive enterocytes and numerous goblet cells. While the general mucosal architecture is preserved, goblet cell mucins appear pale or unstained, and collagen and muscle fibers in the lamina propria are difficult to differentiate due to the uniform eosinophilic background. (**B**) Pentachroma O-H staining of a serial section from the same tissue level highlights five distinct histological components: acidic mucins in goblet cells are stained turquoise–blue (Alcian Blue), collagen fibers in the lamina propria appear red (acid fuchsin), smooth muscle and erythrocyte cytoplasm are yellow (picric acid), elastic fibers are dark–purple (resorcin–fuchsin), and cell nuclei are distinctly blue–black (Mayer’s hematoxylin). This multicolor stain provides enhanced contrast and facilitates clearer identification of mucin content, connective tissue structure, and vascular components within a single section. Scale bar: (**A**)—0.060 mm; (**B**)—0.060 mm.

Figure 8A shows an ileum section in a standard H&E stain (200× magnification). The general villous architecture was evident, being well preserved in H&E: numerous goblet cells can be seen interspersed among the absorptive cells. However, as is typical with H&E, the goblet cell mucin droplets were only faintly discernable—these intracytoplasmic inclusions appeared extremely pale (in a light white-to-gray faint hue), or nearly chromophobic (i.e., completely unstained). Indeed, with conventional H&E, most ileum mucosubstances will not pick up either dye, being usually washed out and lost during processing, thus making goblet cells much more difficult to distinguish within the mucosa, i.e., solely by their weakly contoured shape. The lamina propria in the H&E section was rendered in a uniform eosinophilic pink tinctoriality, whereas collagen fibers, smooth muscle, and other extracellular components all blended into similar shades of pink to purple. This may make it challenging, at times, to distinguish collagenous stroma from smooth muscular fibers, as both take on a similar pink hue against the uniform background; or to identify elastic fibers at all, as they are not specifically stained by H&E. In essence, H&E provided an excellent overview of morphology but limited specificity and biochemical insights: for instance, fibrosis in the lamina propria would appear as pink fibrilarry material but without any clear indication that it is in fact collagen, and/or goblet cell counts could be underestimated due to their mucin droplets looking like empty vacuoles.

Conversely, the Pentachroma O-H section (Figure 8B)—taken from a serial adjacent section of the same tissue sample—presented the ileum mucosa in a far more resolved fashion. Herein, five distinct histological components were each simultaneously rendered in a unique individual tinctoriality: (1) goblet cell acidic mucins were stained a conspicuous turquoise–blue (Alcian Blue-positive), immediately highlighting their distribution and abundance within the epithelial mucosa; (2) collagen fibers throughout the villous core (scarce in the lamina propria) and submucosa (predominant) were stained distinctly red (by Van Gieson’s acid fuchsin); (3) adjacent smooth muscle fibers (for example, within the muscularis mucosae or blood vessel walls), as well as erythrocytes’ cytoplasm (more intensely than muscle), were highlighted in a range of bright yellow-to-orange tones; (4) elastic fibers, essentially undetectable on H&E, were readily identifiable as thin fine black–purple strands (resorcin–fuchsin), interwoven with submucosal collagen and/or distributed around blood vessels; and (5) all cell nuclei were counterstained in a deep blue–black (by Mayer’s hematoxylin), comparable to conventional H&E in clarity, thus providing context and allowing identification of lymphocytes, enterocytes, endothelial cells, and others.

Overall, the improvement in contrast was immediately apparent when compared to H&E: mucin-filled goblet cells that were barely perceptible as pale vacuoles in H&E now stood out in blue against the yellow-stained enterocyte cytoplasm, allowing easy quantification of goblet cell density and mucin distribution. Collagen deposits in the lamina propria and submucosa, which were indistinguishable from smooth muscle on the H&E, were now clearly red and could be differentiated from the thin strands of smooth muscle (stained yellow) that weave through the stroma. Elastic fibers, invisible in Figure 8A, were unambiguously identified in Figure 8B as black threads, indicating the presence and organization of elastic tissue in the intestinal wall.

To sum up, Figure 8 encapsulates these aforementioned differences, with panel (A) illustrating the limited staining profile of H&E, and panel (B) highlighting how Pentachroma O-H clearly distinguishes between acidic mucins, connective tissue fibers (collagen and elastin) and cellularity, smooth muscle, and erythrocytes. This multicolor stain provided markedly enhanced contrast and the ability to discern subtle histological features that were missed by H&E. For example, mild increases in submucosal collagen deposition (early fibrosis) and delicate elastic fiber networks should be far more conspicuous on Pentachroma O-H-stained sections than on the corresponding H&E, underscoring the promising diagnostic advantage of our histochemical combined staining method in detecting and evaluating fine architectural details of human ileum tissue samples.

## 3. Discussion

In this study, we introduced Pentachroma O-H, an original pentachromatic histological staining method, and demonstrated its ability to concurrently visualize mucins, connective tissue fibers, smooth muscle, and nuclei with high contrast. The results on healthy ileum tissue highlight that this single method can provide equivalent or superior information to several classical special histochemical stains used separately. In the Pentachroma O-H stain, acidic mucins (alcianophilic substances) are clearly marked in turquoise–blue, akin to an Alcian Blue pH 2.5 stain [10], collagen fibers are labeled in red and muscle in yellow as in a van Gieson or trichrome stain [7,8,13], and elastic fibers are rendered dark purple/black, as in other dedicated elastin stains [13]. The inclusion of a nuclear counterstain ensures that cellular details and tissue architecture are still easy to orient. Overall, Pentachroma O-H merges the capabilities of multiple histochemical techniques into one workflow, in an attempt to address the need for improved tissue visualization in gastrointestinal diagnostic histology.

The potentially enhanced diagnostic precision afforded by Pentachroma O-H stems from its multicolor visualization of tissue components that are often key in pathology. For example, gastrointestinal pathologists frequently assess both mucin content and fibrous tissue status: goblet cell mucin depletion is a hallmark of conditions like chronic ulcerative colitis, while collagen deposition in the lamina propria can indicate fibrosis or collagenous colitis [19,20]. Traditionally, one might perform an Alcian Blue or AB/PAS stain to highlight goblet cell mucins, and a separate trichrome stain to evaluate fibrosis. Our Pentachroma O-H method reveals both features simultaneously—the goblet cells in blue and the collagen in red—which can facilitate a more immediate and integrated interpretation. In mucinous adenocarcinomas (such as signet-ring cell carcinoma of the stomach), special stains like mucicarmine or AB/PAS have been used to confirm the presence of carcinoma mucins [21]. With Pentachroma O-H, one could easily identify the mucins (turquoise–blue), while at the same time easily observiing fibrosis and desmoplastic stromal responses (red collagen and important lymphoid infiltration—blue–black nuclei with perinuclear clearing), as well as any vascular or elastic component involvement (dark purple/black elastin) in the same section. This comprehensive overview can potentially improve the accuracy of tumor diagnosis and the assessment of tumor microenvironment without toggling between slides.

Beyond gastrointestinal pathology, the ability to clearly visualize elastic fibers and collagen together, at the same time, on one single slide, should also be particularly useful in cardiovascular and pulmonary pathology. For instance, in arteriosclerotic lesions or vasculitis, pathologists particularly examine parietal elastic lamellae integrity and new collagen deposition [22]. Movat’s pentachrome is sometimes requested in such cases, specifically because it differentiates elastin, mucin, fibrin, and collagen so vividly [16,23]. Our Pentachroma O-H achieves a similar outcome, yet with a simpler, more efficient protocol: elastic laminae are clearly stained black to dark purple, while any collagen in the vessel wall or intima will be bright red. Conversely, in pulmonary biopsies, our original method could analogously help identify elastin breaks in emphysema or fibrosis in idiopathic pulmonary fibrosis, differentially highlighting elastic fiber networks versus collagenous scars. The simultaneous differentiation of these components can reveal subtle interrelationships in tissue organization. Indeed, prior work has noted that polychromatic stains like Movat’s can reveal early and subtle changes in connective tissues that routine H&E or single-color special stains might not be able to show [24]. In our results, subtle increases in submucosal fibrosis and the presence of elastic fibers were more conspicuous with Pentachroma O-H than on H&E, supporting this notion. Furthermore, the multicolor contrast aids in appreciating the spatial relationships between different tissue elements—for example, one can easily see how mucin pools abut areas of fibrosis, or how elastic fibers are distributed relative to smooth muscle in the muscularis mucosae, which could be diagnostically relevant (such as when distinguishing a mucinous lesion dissecting through the vascular wall versus contained by an elastic lamina).

Pentachroma O-H offers several advantages over traditional stains, as well as a few limitations. Compared to Masson’s Trichrome, which stains muscle and collagen differently but does not identify mucins or elastic fibers, Pentachroma O-H provides a more complete tissue profile. Masson’s stain is extremely useful for fibrosis assessment, and in muscle tissue pathology [8,25], but one would need to apply an additional Alcian Blue or elastin stain to obtain the information that Pentachroma O-H yields in one fell swoop. In essence, Pentachroma O-H covers the use-cases of three main commonly used histochemical stains (a trichrome, Alcian Blue, and an elastin stain) all together. 

As opposed to AB/PAS, which delineates mucin subtypes [26] but does not individually highlight collagen, elastin, or muscle fibers, Pentachroma O-H sacrifices the differentiation of neutral versus acidic mucins (our method targets acidic mucins primarily), in favor of focusing on connective tissue fibers, i.e., the fibrous network architecture. In scenarios where neutral mucins are a focal interest (e.g., certain adenocarcinomas secreting neutral mucins), AB/PAS would still be the method of choice. However, many tissue mucins (intestinal, bronchial, etc.) are usually acidic and thus readily demonstrated by Alcian Blue [27], with Pentachroma O-H being thereby highly effective in most clinical contexts. In fact, we are currently investigating the possibility of further integrating an additional PAS step into the Pentachroma O-H sequence (after Alcian Blue but before hematoxylin), in order to also stain neutral mucins magenta, thus effectively creating a further “hexachrome” stain. This was not explored in our current protocol to avoid over-complicating the staining and color interpretation, but it suggests flexibility for future modification.

Compared to Movat’s pentachrome, the closest existing analog, Pentachroma O-H is streamlined and uses more common reagents. Movat’s stain, especially in its initial format, requires multiple sequential dye baths and differentiation steps (Alcian Blue, alkaline alcohol, iron hematoxylin with iodine, sodium thiosulfate, scarlet–fuchsin, phosphotungstic acid, and saffron) [28]. The use of saffron in Movat’s method to stain collagen yellow is a distinctive, yet highly inconvenient aspect—saffron (a natural dye from Crocus sativus) can be quite expensive and of variable quality [29]. In Pentachroma O-H, we achieve collagen staining via the classic van Gieson method (acid fuchsin yielding red collagen [30]), which avoids the need for saffron, while still providing clear contrast between collagen and muscle fibers. The trade-off is that collagen is red (similar color family as muscle’s yellow to orange) rather than a completely different color (yellow) to other targeted components, as in Movat’s. Even so, our current results indicate that these tinctorialities (red versus yellow) were consistently specific and sufficiently distinct to accurately distinguish between collagen and muscle, especially under proper differentiation. 

Importantly, the staining time and complexity are significantly reduced in Pentachroma O-H. Our protocol can be completed in roughly 45 min (after deparaffinization), whereas a traditional Movat’s generally takes well over two hours and often requires careful and very specialized technician oversight to achieve consistent results [14,28,31]. Russell’s modified Movat’s method brought the staining time down to just over one hour by simplifying steps [15]. Even so, our original Pentachroma O-H protocol is still significantly more efficient, technically, as well as time-wise. We also found that Pentachroma O-H is quite reproducible—the use of stable solutions like Mayer’s hematoxylin and commercial van Gieson reduces variability. In contrast, Movat’s hematoxylin step (Verhoeff’s solution) uses an iron hematoxylin that must be prepared fresh and carefully timed to avoid over- or under-staining; similarly, the differentiation with phosphotungstic acid and the proper endpoint for saffron can be somewhat subjective, potentially affecting consistency [14,32]. By using Weigert’s resorcin–fuchsin for elastin and a straightforward van Gieson counterstain, Pentachroma O-H may be considered more technically forgiving and/or easier to implement in a routine lab setting.

One limitation of Pentachroma O-H relative to Movat’s is the handling of fibrin. Movat’s pentachrome explicitly stains fibrin (and muscle) a bright red, distinct from the yellow of collagen [14,33], aiding in identification of fibrin deposits (e.g., in thrombi or acute inflammation). In our Pentachroma O-H method, fibrin would be stained by the picric acid from van Gieson’s solution, thus appearing yellow (similar to muscle) [34]. This means that Pentachroma O-H does not specifically distinguish fibrin from smooth muscle fibers, erythrocytes, and/or other cytoplasmic elements; fibrin would likely blend with the yellow background. If fibrin demonstration is needed, one might supplement Pentachroma O-H with a fibrin-specific stain (Martius Scarlet Blue, or Phosphotungstic Acid Hematoxylin) on a separate slide [35] or modify the protocol to include a different coloring agent for fibrin (a subject of future method development). Nonetheless, for many diagnostic purposes (outside of acute thrombi), fibrin is less commonly the sole focus compared to mucin, collagen, and elastin, so this limitation is in fact a minor one in routine use.

Another consideration is the metachromatic substrates that toluidine blue would normally highlight (mast cell granules, cartilage matrix). In Pentachroma O-H, mast cell granules, which contain heparin (acidic), would theoretically take Alcian Blue and appear blue; this should still be effective for identifying mast cells, notwithstanding toluidine blue’s purple–red metachromasia being a more classic identifier [12,36]. Cartilage (rich in glycosaminoglycans) would also stain with Alcian Blue in our method (blue ground substance), and collagen in cartilage would be red, so cartilage would likely appear as a purple mix of blue and red—potentially recognizable, but not as single-tone as toluidine blue’s purple. Thus, Pentachroma O-H can reveal cartilage and mast cells, but interpretation may require awareness that colors can mix when components co-localize (i.e., blue Alcian staining + red Van Gieson’s collagen in cartilage yielding a purple composite). Conversely, this limitation is inherent to any multi-stain: overlapping tissue components might produce intermediate combined hues. In our experience with the ileum (which contains no cartilage and only sparsely scattered mast cells), this was not a significant issue. In tissues with more complex overlaps, the pathologist’s understanding of the staining chemistry would guide interpretation (much as one learns to interpret Movat’s or trichrome results when colors overlap).

From a workflow integration perspective, Pentachroma O-H is designed to fit into routine laboratory operations. All reagents used are standard in histology labs or easily obtainable (Alcian Blue, Mayer’s hematoxylin, etc., from major suppliers). The procedure does not require any special equipment beyond what is typical for other commonly used stains. It can be performed manually, or the protocol could be adapted to an automated staining platform that handles special stains. The number of steps is comparable to performing two separate special stains sequentially; thus, a lab already performing Alcian Blue and trichrome stains should be capable of implementing Pentachroma O-H without significant additional effort. One practical point is that sections should be well adhered to slides (we used pre-treated charged slides) because the multiple aqueous and alcoholic steps could cause poorly adhered sections to detach—this is standard practice for any multi-step stain or when using solutions like picric acid. In terms of safety and cost, Pentachroma O-H uses picric acid (in van Gieson), which is an explosive hazard in dry form but safe in solution [37]; laboratories already using picric acid fixatives or van Gieson will be familiar with its handling. Seeing as no particularly exotic or expensive dyes are used, the cost per slide for Pentachroma O-H is expected to be lower than the sum of its individual constituents, i.e., it could even be cost-saving by reducing the number of separate stains ordered.

The development of digital pathology and artificial intelligence has created new opportunities for standardized staining protocols. Recent advances have demonstrated the potential for deep learning algorithms to achieve diagnostic accuracy comparable to pathologists [38,39]. However, these systems require consistent, high-quality staining that reveals multiple tissue components simultaneously. Pentachroma O-H could serve as an ideal ground truth for algorithm training, as it provides five distinct colors that can be easily segmented and analyzed by automated systems.

In the current era, IHC is often used to identify specific molecular markers in tissues, typically yielding brown (DAB chromogen) staining on an H&E or hematoxylin counterstain. A downside of routine IHC is the limited color range (usually brown DAB and blue hematoxylin), which can obscure the tissue’s general morphology [40]. Polychromatic stains like Pentachroma O-H could play a complementary role here. For example, one could perform an immunostain (with DAB, brown) and use a pentachrome counterstain instead of the usual hematoxylin. Prior research has explored this: Petrovic et al. developed a modified Movat’s pentachrome as a counterstain for IHC to better visualize the tissue context around IHC-positive cells [16,28]. They noted that Movat’s original method was less suitable with DAB because the colors could be similar, but by tweaking the stain (e.g., altering the fuchsin step), they later achieved stronger color contrast with the brown DAB product [41]. In our Pentachroma O-H, the presence of multiple bright colors (blue, red, yellow) could likewise provide a vibrant background to a brown or red IHC chromogen, aiding in simultaneously evaluating the immune-labeled cells, as well as the state of the stroma or mucins around them. This could be particularly useful in research settings examining tumor stroma interactions, or in diagnostic cases like distinguishing whether a tumor has invaded through an elastic layer (a Pentachroma O-H could show the elastic layer and an immunostain for tumor cells in one field). While we did not explicitly test Pentachroma O-H as an IHC counterstain here, the concept is promising and aligns with the idea of using multicolor histochemistry to complement immunohistochemical specificity [41,42].

Aside from the previously mentioned lack of neutral mucin and fibrin distinction, one further limitation to acknowledge is that pathologists must become familiar with the Pentachroma O-H color scheme to interpret slides correctly. This is a short learning curve similar to that for Movat’s pentachrome or any other special stain. Each colored element must be recognized in context—for instance, knowing that blue indicates acidic mucin or the cartilage matrix, black indicates elastin or nuclei, etc. There is minimal risk of confusion, as the colors are quite distinct, but overlapping components could theoretically yield mixed colors (e.g., structures rich in both mucin and collagen might appear blue–purple or purple–red). Control slides and growing experience will mitigate this issue. Another potential limitation is that if a case requires quantification of a particular component (e.g., fibrosis grading), the presence of multiple colors might complicate digital image analysis compared to a single-color stain for that component. However, modern image analysis software could be trained to detect the specific color ranges corresponding to collagen, for instance, in Pentachroma O-H sections.

Finally, it should be noted that Pentachroma O-H is intended for morphological histology and is not a substitute for special histochemical reactions that identify specific biochemical constituents beyond those targeted (for example, it will not identify iron, microbes, or amyloid—those still need their respective iron stains, Gram/Ziehl–Neelsen, Congo red, etc.). It is specifically tailored to general tissue architecture. In that domain, our findings suggest it can reduce the need for multiple sequential stains, thereby saving tissue and time. However, this study is limited by its single-patient sample source and lack of statistical validation. Future work must include multiple patients, diverse tissue types, and quantitative assessment of staining reproducibility.

## 4. Materials and Methods

### 4.1. Study Design

This original histochemical staining method development study was approved by the Research Ethics Committee of the Victor Babes University of Medicine and Pharmacy Timisoara (72/2 November 2024). Healthy human ileum tissue was obtained during clinical surgical practice, from a patient undergoing palliative urinary diversion (Bricker continent ileum pouch) for advanced cervical cancer, as a result of an initially ischemic mechanic ileum anastomosis, which required further excision and revision. The surgery was performed by our urologist (D.N.) and general surgeon (C.T.) coauthors, within the Department of General Surgery II, at Timisoara County Emergency Clinical Hospital. Written informed consent has been obtained from the patient for further histological processing of the ileum specimen and to publish the results of our combined staining investigation.

This case was ideal for the aim of our current proof-of-concept descriptive histology investigation: to define the original protocol and descriptively document the emerging morphological particularities of our combined histochemical stain Pentachroma O-H, developed specifically for enhanced gastrointestinal pathology assessment, using healthy human ileum tissue samples. After processing, 15 FFPE samples were sectioned (4 serial sections from each block), stained sequentially (one in H&E, the next in Pentachroma O-H), and assessed microscopically for inclusion criteria conformity.

Inclusion criteria for Pentachroma O-H staining were as follows: normal overall histology on H&E, intact mucosal architecture, adequate tissue sample size (>1 cm^2^), and proper fixation. Exclusion criteria were as follows: autolysis, extensive hemorrhage, and inadequate fixation. Finally, the study population comprised 60 serial section slides: 30 control H&E stains and 30 adjacent Pentachroma O-H stains of normal ileum tissue.

### 4.2. Tissue Sample Processing

The fresh surgical specimen was fixed in 10% neutral buffered formalin for 48 h, then macroscopically assessed, and sampled (15 distinct serial samples, avoiding the mechanical anastomosis area and the excision margins) by a senior pathologist (F.Z.), so that these healthy ileum tissue samples could thereafter be processed routinely to paraffin. Paraffin embedding and sectioning were performed using standard histology equipment. Briefly, tissue cassettes were processed in a Thermo Shandon Citadel 2000 automated tissue processor (Thermo Fisher Scientific, Waltham, MA, USA) through graded alcohols, clearing in xylene, and infiltration with paraffin wax. Processed specimens were embedded in paraffin using a Thermo Shandon Histocentre 3 embedding station.

Serial sections (3 μm thickness) were sliced on a Leica HistoCore Multicut rotary microtome (Leica Biosystems, Deer Park, IL, USA) for parallel staining comparisons—one set of sections was stained with the routine H&E method (30 conventional slides), and corresponding adjacent sections (30 slides) were stained with the original Pentachroma O-H technique described below. To ensure tissue adherence throughout the multiple staining steps, sections were floated on a 45 °C water bath and then mounted on pretreated (Mayer’s albumin and a drop of distilled water), positively charged glass slides (Leica X-tra Adhesive, Leica Biosystems, Deer Park, IL, USA).

### 4.3. Staining Protocols

After routine processing and sectioning (as described above), tissue slides were assessed for conformity and then stained with our newly developed Pentachroma O-H technique—an original five-color histochemical staining method, performed semi-manually, at room temperature (~22 °C). Preliminary thermal treatment (60 min at 59 °C), deparaffinization (by immersion in xylene: 3 changes, 5 min each), and rehydration (graded ethanol series: 100% ethanol (2 changes), 95%, 70%, and then running tap water, 1–2 min each), bringing sections to a final distilled water bath, were all carried out on an automated slide stainer (Leica Autostainer XL, Leica Biosystems, Deer Park, IL, USA), according to standard protocols, using analytical-grade chemicals. 

Thereafter, all subsequent staining steps were performed by hand, using readily available histological reagents for microscopy, obtained as ready-to-use solutions from reputable commercial sources, as detailed below in Table 1:

The Pentachroma O-H staining workflow was elaborated and implemented as follows:

1. Mucin stain: Slides were immersed in the Alcian Blue solution, pH 2.5 (see Table 1), for 30 min. This step stains acidic mucins turquoise–blue by forming insoluble complexes (“monastral blue”) with acid mucopolysaccharides. After staining, sections were rinsed gently in distilled water to remove excess reagent solution, i.e., 2 brief rinses. Notably, no pretreatment with diastase was performed, so any glycogen present would also later react with Schiff’s reagent in PAS if it were applied; however, in this protocol PAS was not used, focusing on acidic mucins.
2. Elastin Stain: Sections were then incubated in Weigert’s resorcin–fuchsin working solution (resorcinol and basic fuchsin with ferric chloride mordant) for 10 min. This stain selectively dyes elastic fibers a deep purple-to-black color, by the formation of hydrophobic dye complex deposits onto elastic fibers due to electrostatic attraction (negatively charged elastin microfibrils) (see Table 1). After staining, the slides were differentiated by immersing in 95% ethanol until the background turned light and only elastic fibers remained darkly stained (approximately 30–60 s; differentiation was monitored microscopically). The differentiation step is critical because Weigert’s elastic stain is not absolutely specific and can weakly color other structures like collagen if not controlled. Once adequate differentiation was achieved—elastic fibers appearing sharply dark on a nearly colorless background—sections were rinsed in running water for 1 min. At this stage, elastic fibers are permanently stained and will retain a dark purple–black hue through subsequent steps, whereas collagen and other tissues have little to no residual color.
3. Nuclear Stain: Onward, using a dropper, slides were covered in Mayer’s hematoxylin and allowed to stain the cell nuclei for 2 min. This alum–hematoxylin imparts a deep blue–purple nuclear coloration. Subsequently, sections were rinsed in tap water for 1 min. To achieve the characteristic crisp blue nucleus, slides were then covered in the alkaline bluing buffer solution for 10 s, converting the hematoxylin to its blue-colored insoluble form (see Table 1). A further 1-min rinse in running tap water ensured complete bluing and removal of excess reagent. Alternatively, bluing can also be accomplished by a longer tap water rinse alone, since tap water’s slight alkalinity will also gradually blue hematoxylin; in our protocol, we used a standardized commercial reagent for consistency and speed.
4. Counterstain: Finally, sections were immersed with Van Gieson’s picric acid–fuchsin solution for 1 min. This is a two-component acidic dye solution in which acid fuchsin selectively stains collagen fibers red, and picric acid stains muscle, cytoplasm, and red blood cells varying shades of yellow. During this step, the previously hematoxylin-stained nuclei are relatively resistant to the acidic dye (especially since alum-hematoxylin is partly resistant; an iron hematoxylin would be even more resistant). The elastic fibers, already colored purple–black by resorcin–fuchsin, largely remain dark; picric acid may impart a slight yellow tint to them, but their intense purple–black from the elastin stain is mostly retained. After 1 min in Van Gieson’s solution, the slides were rinsed very briefly in distilled water (no more than 2–3 s, as prolonged water exposure can diffuse picric acid–bound dye).
5. Dehydration and Mounting: The sections were then quickly dehydrated in two 10 s changes of 100% ethanol (the picric acid differentiates slightly during dehydration, i.e., a quick dehydration minimizes picric acid leaching) and cleared in xylene (2 changes, 30 s each) and cover-slipped with a resinous mounting medium.

Control sections known to contain the target components (e.g., skin or vessel wall for elastin, goblet-cell-rich colon for mucin) were run in parallel to verify staining quality. No antigen retrieval or special pretreatments were required aside from those stated. Remarkably, the entire Pentachroma O-H protocol can be completed in 45 min (surely under 1 h), much quicker than the intricate classical Movat’s pentachrome.

For comparison, automated conventional H&E staining (Leica Autostainer XL, Leica Biosystems, Deer Park, IL, USA) was performed on adjacent ileum sections (to the aforementioned Pentachroma O-H slides), using a standardized protocol. Briefly, slides were stained in Harris hematoxylin (5 min); differentiated and blued by running water rinses; and counterstained in Eosin-Y solution (2 min). This provided baseline histology against which the original Pentachroma O-H results were evaluated.

### 4.4. Image Acquisition and Analysis 

Stained sections were examined using a Leica DM2500 microscope (Leica Biosystems, Deer Park, IL, USA) equipped with a digital camera system. Images were captured at magnifications of 50×, 100×, 200×, and 400× to document overall tissue architecture and cellular details. White balance and exposure settings were standardized across all image acquisitions.

No quantitative image analysis was performed in this study, but qualitative comparisons between H&E and Pentachroma O-H were documented, as described below. The Pentachroma O-H stained sections were assessed for the color assignment, contrast, and specificity of staining in different intestinal tissue constituents (mucin in goblet cells, collagen in submucosa, elastic fibers in vessel walls, muscle in muscularis propria, etc.) and then compared directly to the appearance of those constituents in the serial section H&E morphology. Descriptive figure legends were prepared to document the expected appearance of key features in both staining methods. Figure images were assembled with minimal digital processing (only global adjustments to brightness/contrast applied equally across the image). The Pentachroma O-H colors were observed to be stable and did not show significant fading or bleed when sections were stored for several months.

## 5. Conclusions

We have developed and characterized the Pentachroma O-H staining method as an original approach to simultaneously visualize five key tissue elements: acidic mucins (blue), elastic fibers (black–purple), collagen (red), smooth muscle and erythrocyte cytoplasm (yellow), and nuclei (blue–black). This single-slide pentachromatic technique provides a comprehensive morphological overview that previously required multiple different stains. In ileum tissue, Pentachroma O-H markedly improved the contrast and detectability of goblet cell mucins, connective tissue fibers, smooth muscle, and vascular elastic laminae, when compared to routine H&E, allowing finer structural details and layer distinctions to be observed. The use of widely available reagents and a streamlined protocol means that Pentachroma O-H can be readily adopted in histopathology laboratories, potentially as part of the routine special stain arsenal. Major advantages of this method include enhanced diagnostic information yield (especially for diseases involving mucin and connective tissue changes), preservation of tissue resources (one slide doing the work of two or three), and compatibility with other techniques (it could complement IHC analyses by providing rich background detail).

In summary, Pentachroma O-H addresses an unmet need for an efficient, multi-component histological stain. It combines the strengths of Alcian Blue, elastin stains, and trichrome stains into one procedure, producing a final histochemically stained slide in which the interplay between covering epithelium, mucins, stroma, and vasculature can all be appreciated at a glance. We believe this method will be valuable for diagnostic histopathology—for example, in gastrointestinal biopsies, connective tissue disorder biopsies, and neoplastic tissue analysis—wherever improved visualization of tissue architecture is desired. Future work may explore slight modifications (such as adding a PAS step for neutral mucins or image analysis applications) and broader validation across tissue types and pathological conditions. Based on our findings, Pentachroma O-H emerges as a powerful addition to the histological toolkit, uniting multiple colors and tissue constituents in service of clearer, more informative microscopy.

In gastroenterology, this original pentachrome stain offers pathologists a richer view of the tissue microenvironment, potentially enhancing diagnostic accuracy and efficiency. The clinical significance of improved tissue contrast—as provided by Pentachroma O-H—lies in more confident interpretation of complex lesions, better correlation of multi-faceted pathological changes, and, ultimately, improved patient diagnosis and care.

## Figures and Tables

**Table 1:** Summary of reagents used in the original Pentachroma O-H staining method.

Reagent	Supplier (City, Country)	Description/Specificity
Alcian Blue solution, pH 2.5	Merck KGaA (Darmstadt, Germany)	A cationic copper–phthalocyanine dye that binds to anionic sulfated and carboxylated mucosubstances (acid mucopolysaccharides) via salt linkages, staining acidic epithelial and connective tissue mucins a turquoise-blue color.
Elastin solution acc. to Weigert *	Merck KGaA (Darmstadt, Germany)	An elastin-staining resorcin–fuchsin solution, which deposits a deep purple–black hydrophobic dye complex onto elastic fibers by electrostatic attraction to their negatively charged elastin microfibrils; i.e., the classical Weigert’s stain.
Mayer’s Hematoxylin	Dako, Agilent Technologies (Glostrup, Denmark)	A mercury-free, alum–hematoxylin formulation for nuclear staining, imparting a dark purplish-blue color to cell nuclei. Because alum–hematoxylin is not resistant to acidic solutions, a separate bluing step is used to stabilize the blue color.
Bluing Reagent	Dako, Agilent Technologies (Glostrup, Denmark)	An alkaline buffer that converts the initial red color of soluble hematoxylin to an insoluble blue form, thereby providing enhanced nuclear contrast.
Picrofuchsin solution acc. to van Gieson **	Merck KGaA (Darmstadt, Germany)	Acts as a connective tissue counterstain, in which small picric acid molecules penetrate muscle and cytoplasm (staining them yellow), while larger acid fuchsin molecules bind selectively to collagen fibers (staining them red). This sharply contrasts collagen vs. smooth muscle and other cytoplasmic elements.

*—Reagent 3 of the Elastica van Gieson kit; **—Reagent 4 of the Elastica van Gieson kit.

## Data Availability

The original contributions presented in this study are included in the article. Further inquiries can be directed to the corresponding author(s).

## Abbreviations

The following abbreviations are used in this manuscript:

AB/PAS	Alcian Blue–Periodic Acid Schiff
DAB	3,3′-Diaminobenzidine
FAE	Follicle-Associated Epithelium
FFPE	Formalin-Fixed, Paraffin-Embedded
GALT	Gut-Associated Lymphoid Tissue
H&E	Hematoxylin and Eosin
IHC	Immunohistochemistry
PAS	Periodic Acid–Schiff
